# MRI-Driven PET Image Optimization for Neurological Applications

**DOI:** 10.3389/fnins.2019.00782

**Published:** 2019-07-31

**Authors:** Yuankai Zhu, Xiaohua Zhu

**Affiliations:** Department of Nuclear Medicine, Tongji Hospital, Tongji Medical College, Huazhong University of Science and Technology, Wuhan, China

**Keywords:** positron emission tomography (PET), magnetic resonance imaging (MRI), neuroimaging, motion correction (MC), partial volume effect (PVE), image-derived input function (IDIF), multimodal imaging

## Abstract

Positron emission tomography (PET) and magnetic resonance imaging (MRI) are established imaging modalities for the study of neurological disorders, such as epilepsy, dementia, psychiatric disorders and so on. Since these two available modalities vary in imaging principle and physical performance, each technique has its own advantages and disadvantages over the other. To acquire the mutual complementary information and reinforce each other, there is a need for the fusion of PET and MRI. This combined dual-modality (either sequential or simultaneous) could generate preferable soft tissue contrast of brain tissue, flexible acquisition parameters, and minimized exposure to radiation. The most unique superiority of PET/MRI is mainly manifested in MRI-based improvement for the inherent limitations of PET, such as motion artifacts, partial volume effect (PVE) and invasive procedure in quantitative analysis. Head motion during scanning significantly deteriorates the effective resolution of PET image, especially for the dynamic scan with lengthy time. Hybrid PET/MRI device can offer motion correction (MC) for PET data through MRI information acquired simultaneously. Regarding the PVE associated with limited spatial resolution, the process and reconstruction of PET data can be further optimized by using acquired MRI either sequentially or simultaneously. The quantitative analysis of dynamic PET data mainly relies upon an invasive arterial blood sampling procedure to acquire arterial input function (AIF). An image-derived input function (IDIF) method without the need of arterial cannulization, can serve as a potential alternative estimation of AIF. Compared with using PET data only, combining anatomical or functional information from MRI for improving the accuracy in IDIF approach has been demonstrated. Yet, due to the interference and inherent disparity between the two modalities, these methods for optimizing PET image based on MRI still have many technical challenges. This review discussed upon the most recent progress, current challenges and future directions of MRI-driven PET data optimization for neurological applications, with either sequential or simultaneous acquisition approach.

## Introduction

Positron emission tomography (PET), a noninvasive imaging modality, presents the physiological and pathophysiological process at molecular level by using various positron tracers (Jones and Rabiner, 2012). This powerful imaging technique is considered to be ideally suitable for monitoring molecular events in the early course of neurological disease, as well as during the course of medical treatment (Gambhir, 2002). However, PET imaging can only offer relatively poor anatomical information, spatial and temporal resolution, which are regarded as its major deficiencies. On the other hand, magnetic resonance imaging (MRI) provides a higher quality of soft tissue resolution, and has higher temporal resolution. Thus, MRI is considered to be an excellent structural and functional imaging modality, which also has obvious advantages in neurological applications (Thomalla et al., 2018). With the development of technology, MRI-based functional imaging including functional MRI (fMRI), magnetic resonance spectroscopy (MRS), diffusion weighted imaging (DWI), and perfusion weighted imaging (PWI), have been introduced into research and clinic extensively.

Although these two available modalities vary in imaging principle and physical performance, each technique has its own advantages and disadvantages over the other. To acquire the mutual complementary information and reinforce each other, there is a need for the fusion of PET and MRI. This combined dual-modalities (either sequential or simultaneous) could generate preferable soft tissue contrast of brain tissue, flexible acquisition parameters and minimized exposure to radiation (Musafargani et al., 2018). Fusion of PET and MRI images acquired at different time points from separated devices, has been performed routinely for brain imaging in a number of clinical centers (Zhu et al., 2017b; Ding et al., 2018). However, it must be pointed out that the physiological or mental state studied, respectively, by PET and MRI may differ in varying levels during the two separated imaging sessions. Since patient’s condition could change between the two sessions of scanning, the confidence of findings from two imaging modalities relating to each other will be questioned (Queiroz et al., 2018). Brain imaging was destined to be the first applications of hybrid (simultaneous) PET/MRI system, which lead to real multiparametric functional analysis by using the two powerful modalities. Currently, studies on distinctive practicability of hybrid PET/MRI are rapidly appearing for neurological applications (van Bergen et al., 2018). This review discussed upon the most unique superiority of PET/MRI, which is mainly manifested in MRI-based improvement for the inherent limitations of PET.

Due to the sequential imaging design of PET/MRI study, head motion between the two acquisitions or within PET scanning could cause errors, especially for the dynamic scan with lengthy time. Hybrid PET/MRI device can offer motion correction (MC) for PET data through MRI information acquired simultaneously (Chen et al., 2018). As MRI could offer better soft tissue resolution than CT, fusion of PET and MRI has more pulling power and results in combining excellent structural information with functional PET data. These MRI-based structural information could be used for not only providing anatomical reference, but also addressing spatial resolution limitations of PET imaging. Regarding the partial volume effect (PVE) associated with limited spatial resolution, the process and reconstruction of PET data can be further optimized by using MRI information acquired either sequentially or simultaneously. The quantitative analysis of dynamic PET data relies upon an invasive arterial blood sampling procedure to acquire arterial input function (AIF). An image-derived input function (IDIF) method without the need of arterial cannulization, can serve as a potential alternative estimation of AIF. Compared with using PET data only, combining anatomical or functional information from MRI for improving the accuracy in this approach has been demonstrated. Yet, due to the interference and inherent disparity between the two modalities, these methods still have many technical challenges. This review discusses upon the most recent progress, current challenges and future directions of MRI-driven PET data optimization for neurological applications, with either sequential or simultaneous acquisition approach.

## MRI-Based MC

Since image quality of PET highly depends on the counts of photons captured within the field of view, data acquisitions of PET commonly require several minutes per position or per frame. The intrinsic spatial resolution of clinical PET scanners is considered to be in the range of 3∼5 mm, which may not be achieved due to either involuntary or voluntary motion during PET scanning (Blume et al., 2010; Kustner et al., 2017). The inevitable motion in varying degrees includes two distinct categories, nonrigid (deformable) and rigid motion. Both cardiac and respiratory motion can be regarded as deformable motion (Bousse et al., 2016). The head movement, a type of rigid motion, is mainly due to the translation or rotation of head during PET study. The effective resolution of PET image could be significantly deteriorated by such motion artifacts (Ouyang et al., 2013).

In the clinical practice of brain PET imaging, the visual evaluation of static PET images is generally not apparently influenced by slight head motion. However, such blurring could induce an unrealistic picture of PET tracer distribution. Furthermore, the impaired image quality could give rise to bias and increased variability in the statistical comparison of group studies (Herzog et al., 2005). Despite the fact that not all the head of patients with neurological disorders move more than healthy subjects, a greater percentage of these patients are tend to experience head motion (Ikari et al., 2012). Apart from the introduced blurring and image quality degradation, head motion also impedes the co-registration of these two modalities and further induces emission-attenuation mismatch. Therefore, head motion should be corrected before or within data reconstruction in order to obtain precise lesion identification and data quantification.

Although the dedicated head holders have been designed to minimize head motion during PET and/or MRI scanning, the excessive restriction generally reduces the comfort level (Lalush, 2017). Head holders and earphones used in the hybrid PET/MRI scanner will attenuate the PET signal as well. In addition, some subjects in neurological research may not be able to remain absolute still in spite of wearing head holder.

### Approaches for MC

MC algorithm attempting to compensate for head movement is another feasible strategy, which can be divided into two groups. The first group of this category is frame-based MC (FBMC) methods, in which the head motion is corrected by co-registering a sequence of PET images to the reference template (Montgomery et al., 2006). Nevertheless, FBMC methods are considered to be reliable only if the variance among various frames is less than 5 mm (Jin et al., 2013). Although commercially available fusion software performs well for co-registering separately acquired PET and MRI images, they cannot compensate for the rigid motion presented within frame. Compared to standalone MRI or PET/CT system, movement artifacts introduce a tougher issue in hybrid PET/MRI scanner. Thus, an sensitive and effective technique for motion-detection and MC should be utilized, and the dynamic arrangement of PET frames should achieve a proper compromise between signal to noise ratio (SNR) and MC accuracy (Cabello and Ziegler, 2018). Another group of MC methods for compensating head movement are known as event-based methods, which adjust the raw list-mode PET images such that the realistic coincidence events are restored to the standard coordinate resembling the acquisition in the absence of any head motion (Catana et al., 2011; Keller et al., 2015). This method requires real-time monitor of the head motion during PET imaging, by using either an optical motion tracking system or other simultaneous imaging modality such as MRI with excellent anatomical information (Kyme et al., 2018; Chen et al., 2019).

Real-time head motion tracking system using camera or infrared light makes allowance for the reliability of MRI sequences and PET data with severe head movements (Montgomery et al., 2006; Olesen et al., 2012). This type of MC enables considerable image improvement of the PET data (Marner et al., 2017). Since motion tracking data are intrinsically acquired from optical camera and are transformed into the dynamic frames by using a calibration, deviation of the transformation parameter may ruin the endeavor from head tracking system (Zahneisen et al., 2014). It is difficult to maintain the line-of-sight between the optical markers and detectors due to the limited space in the scanners. In addition, the fixation of the target on head can have several technical issues (Aksoy et al., 2011). During facial movements, it may not hold true that the marker taped to the head has a relatively fixed position to the brain. Friction between posterior head and MRI coil may also slightly shift the relative position between scalp and skull during head motion (Gumus et al., 2015).

MRI provides high brain soft-tissue contrast and temporal resolution, which makes it ideal for demonstrating the dynamic head motion. With the involvement of PET and MRI dual-modalities in brain imaging, MRI-driven MC (MRMC) for voluntary head motion during a lengthy PET study can be crucial in maintaining the alignment between a series of PET and MRI images (Reilhac et al., 2018). Hybrid PET/MRI system allows for acquiring MRI images with dynamically changing anatomy at well as list-mode PET acquisition. The accurate acquisition time of each photon pair is also logged in this procedure. Then, MRMC of PET images refers to the orientation and position changes obtained by simultaneous MRI data, and the motion blurring of PET data can be alleviated by the retroactive correction. Furthermore, the MRMC often applies the deformable models for cardiac or respiratory motion in the thorax, while the rigid models for brain motion in the skull (Catana et al., 2011; Fürst et al., 2015; Manber et al., 2015). Up to now, various MRMC strategies have been applied in the neurologic field, and their feasibility suggested by several proof-of-principle studies (Catana et al., 2011; Ullisch et al., 2012).

### Procedure of MRMC

**FIGURE 1:**
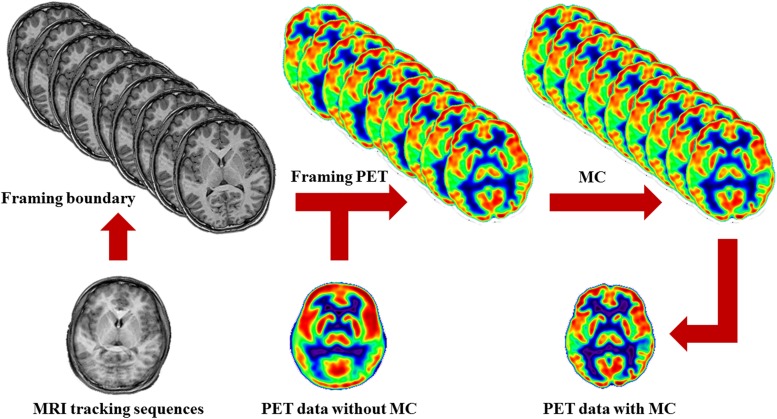
Schematic of MRMC procedures. MRI tracking sequences can be used to monitor the head motion during PET scanning. Each time point when head motion is beyond a default threshold is determined as a symbol of actual movement and a framing boundary for the recombination of the list-mode PET images. By this means, the successive PET raw data are divided into a series of discrete temporal subunits, each of which maintains a specific fixed head pose. Then, the process of MRMC for PET data either within-reconstruction or post-reconstruction is completed by assembling all the framed PET images into a single PET image.

Although MRMC techniques are diverse and complicated, several essential routine procedures are involved (Figure 1). Above all, simultaneous acquisition of fast MRI signals is considered to be the primary premise for MRMC. During the PET scanning, head motion tracking signals using various MRI sequences infer the real-time head movement. The commonly used fast MRI navigator sequences for head motion comprise fast echo planar imaging (EPI) sequences, and active MRI microcoils (Catana et al., 2011; Ullisch et al., 2012; Huang et al., 2014a,b; Keller et al., 2015). MRI navigator volumes can be acquired between other scheduled MRI sequences at multiple time points. At each sample point, a series of navigator volumes are acquired to check the noise and brain motion in the process of navigator-to-navigator registration (Keller et al., 2015). All the navigators acquired intermittently from each sample point are rigidly registered to the first one, and the motion magnitude and direction of each sample point are calculated. Alternatively, applications of image analysis software also allows for the head motion tracking by realigning the series of brain image volumes acquired using EPI sequence with a high temporal resolution of about 2–3 s (Ullisch et al., 2012). This head motion parameter extracted from an amount of EPI could be exploited for correcting both fMRI and PET images. In addition, active MRI microcoils could also be used as fiducial markers (Huang et al., 2014a,b). This approach offers the possibility of tracking the real-time head motion during brain PET acquisition, and improves the temporal resolution compared with other MRMC methods.

As head motion is monitored by repetitive MRI images, the time point when head motion is beyond a default threshold is determined as a symbol of actual movement and a framing boundary for the recombination of the raw list-mode PET images. By this means, the successive PET data are divided into a series of discrete temporal subunits, each of which maintains a specific fixed head pose. This procedure is referred to as PET framing.

Based on the head pose information as mentioned above, the process of PET correction is completed by assembling all the framed PET images into a single PET image. This decisive step may be conducted in the format of either within-reconstruction or post-reconstruction. Regarding to the former, the entire procedures of head motion modeling and PET correction are integrated with the PET image reconstruction (Fürst et al., 2015; Chen et al., 2019). But for the latter, each frame of PET data is reconstructed separately and then realigned into a reference brain template, and summed eventually (Catana et al., 2011). Without loss of PET SNR, significant improvement in lesion detection and reducing artifacts derived from misalignment could be generated by MRMC (Chun et al., 2012; Chen et al., 2019).

## MRI-Based Partial Volume Correction

Spatial resolution signifies the minimum differentiable distance between two points within an image. Various factors, including detector size, positron range, noncollinearity and reconstruction method, contribute to the actual spatial resolution of PET device. In either multidetector or continuous single detectors PET scanners, the dominant factor affecting the intrinsic spatial resolution is the detector size or the number of photons detected, respectively. A positron travels a distance in tissue before annihilation, and the distance relies on the energy of this positron. Therefore, the positron kinetic energy spectrum of specific radioisotope determines the effective positron range, which results in the degradation of spatial resolution (Cal-Gonzalez et al., 2015). In addition, due to the residual momentum of the positron at the annihilation position, two 511 keV photons are emitted at a slight deviation from 180°. This noncollinearity also degrades the spatial resolution of PET scanner, and further deteriorates with larger diameter of detector ring.

Compared with MRI or CT, one of the principal limitations of PET imaging is the relatively low spatial resolution (van der Vos et al., 2017). It is usually referred to as PVE that the relatively limited spatial resolution affects images both visually and quantitatively. Most of the PVE of PET image can be generally regarded as two distinct effects: spill-out of data from inside region and spill-in of data from outside region. Consequently, the edge area of target regions, which has different tissue type or physiological state from adjacent regions, is supposed to be affected by PVE most seriously (Erlandsson et al., 2012). The degree of spill-over between contiguous regions relies on the point-spread function of the medical imaging system, which is customarily modeled as a 3-dimensional (3D) Gaussian function and principally characterized by the extent of full-width at half-maximum (FWHM). Since the cerebral cortex of human is only a few millimeters thick, gray matter suffers from the severe PVE in PET imaging.

Tissue fraction effect (TFE), another component of the PVE, is the result of sampling effect related to the finite voxel size of images. Each single voxel of brain images may contain multiple tissue types [e.g., gray matter, white matter, cerebrospinal fluid (CSF), and blood vessel in brain]. Compared to the spill-over effect between contiguous regions, TFE only accounts for a minor weight of PVE in PET data, but is of greater concern in CT or MRI images. The existing works for TFE correction in emission tomography mostly concentrate on the tissue of lung or myocardium, using density information derived from CT (Pretorius and King, 2009; Holman et al., 2015; Matsunaga et al., 2017). Considering the absence of dedicated MRI-based technique for TFE correction in PET images, this review will not go any further to discuss this subtopic.

The PVE phenomenon can be ameliorated by using several partial volume correction (PVC) methods on PET image (Meechai et al., 2015). Reversing the image degradation is vital for precise quantification of PET data and for avoiding confounding factors derived from PVE. Due to the intrinsic physical limitations of temporal resolution, spatial resolution and noise performance, PET image generally has poorer image quality than MRI. Based on this premise, it is usually supposed that anatomical data derived from MRI can be used to polish up these limitation of PET image (van Bergen et al., 2018). Furthermore, brain is an ideal tissue for the MRI-based PVC by PET/MRI multimodal imaging due to several reasons. First of all, essential co-registration and segmentation of neuroimaging can be performed by using widely available software, such as SPM, MRIcron and so on (Zhu et al., 2017a). These manipulations improve the quantitative errors of PET data induced by poor spatial resolution, using separately or simultaneously acquired MRI images (Meltzer et al., 1996; Erlandsson et al., 2016). Secondly, PET combined with MRI could provide complementary information in neuroscience research (Aiello et al., 2016). And then, since the transformations between PET and MRI images require rigid model only, the processes of co-registration and segmentation for PET/MRI data are feasible and informative.

With regard to the refined definition of specific brain tissue, MRI has high quality of soft tissue contrast, which is especially critical in brain imaging. The anatomical information obtained either sequentially or simultaneously with the acquisition of PET data facilitates the accurate image registration procedure, which is the prerequisite for PVC and other correction techniques. Furthermore, the high quality of MRI soft tissue contrast can give a better segmentation of various tissue types of brain (Meltzer et al., 1999). Anatomical details obtained from the MRI structural image could be used to optimize brain PET in the form of MRI-based PVC.

Before the advent of hybrid PET/MRI system, MRI-based PVC of PET data mainly relies on the separately acquired PET and MRI images (Meltzer et al., 1990). These methods are still available and can be transplanted into the data processing of hybrid PET/MRI system. It is suggested that the process becomes more efficient if these data have been truly simultaneously acquired (Pichler et al., 2010; Musafargani et al., 2018). Combined PET/MRI dual-modalities can offer clear advantages for PVC of PET data, and the degree of adjustment needed for co-registration should be minimal. Therefore, the rising hybrid PET/MRI system could further facilitate the utilization of MRI-based PVC in routine clinical application.

For the present, there is an amount of studies focused on the clinical application of MRI-based PVC for PET images (Ouyang et al., 1994; Reader et al., 2003). As shown in Table 1, the widest utilization of PVC techniques for PET is mainly in the neurological applications (Fessler et al., 1992; Gindi et al., 1993; Ouyang et al., 1994; Su et al., 2017). Being similar to the classification of MRMC, the PVC techniques can also be divided into two main categories, including post-reconstruction methods and within-reconstruction methods (Gutierrez et al., 2012; Chen et al., 2019). Moreover, post-reconstruction methods further fall into two categories: region-based and voxel-based PVC methods. The within-reconstruction methods for PVC can be superior, and considered to be derived from post-reconstruction ones (Nuyts et al., 2005). However, the most PVC methods can provide different amounts of recovery due to different tissues, subject conditions and tracers (Shidahara et al., 2017). Therefore, the utility of specific MRI-based PVC in brain PET imaging should be examined on the basis of an application-specific consideration (Minhas et al., 2018).

**TABLE 1:** Studies on MRI-based partial volume correction for PET neuroimaging.

**References**	**Categories of PVC**	**PVC algorithm**
Müller-Gärtner et al., 1992; Arakawa et al., 2017	Voxel-based PVC	Gray matter PET algorithm determined by MRI
Fessler et al., 1992	Within-reconstruction PVC	Spatially-variant penalized-likelihood method for tomographic image reconstruction based on a weighted Gibbs penalty
Gindi et al., 1993	Within-reconstruction PVC	A model for Bayesian reconstruction with MRI anatomical priors
Ouyang et al., 1994	Within-reconstruction PVC	A modified Bayesian reconstruction called the “weighted line site” method using the prior boundary information
Meltzer et al., 1990, 1996	Voxel-based PVC	Two or four-compartment extension of gray matter PET algorithm
Bowsher et al., 1996	Within-reconstruction PVC	Region labeling approach by assigning higher prior probabilities
Ardekani et al., 1996	Within-reconstruction PVC	Minimum cross-entropy reconstruction
Lipinski et al., 1997	Within-reconstruction PVC	Markov-GEM algorithm and Gauss- EM algorithm
Sastry and Carson, 1997	Within-reconstruction PVC	Tissue composition model using segmented MR images
Rousset et al., 1998; Baker et al., 2017; Fazio et al., 2017; Smith et al., 2017	Region-based PVC	GTM method based on the principles of linear systems and pairwise interaction between identifiable regions
Rangarajan et al., 2000	Within-reconstruction PVC	A novel EM^2^ algorithm
Quarantelli et al., 2004	Voxel-based PVC	Comparison among M-PVEc, MG-PVEc, RPVEc and mMG-PVEc
Baete et al., 2004	Within-reconstruction PVC	Anatomy-based maximum-a-posteriori reconstruction algorithm using segmented MR images
Boussion et al., 2006	Voxel-based PVC	2D mutual multiresolution analysis
Nuyts, 2007	Within-reconstruction PVC	MAP reconstruction using mutual information and joint entropy to define anatomical priors
Tang and Rahmim, 2009	Within-reconstruction PVC	One-step-late MAP algorithm with the joint entropy
Shidahara et al., 2009; Grecchi et al., 2017	Voxel-based PVC	The synergistic use of functional and structural data based on the multiresolution property of the wavelet transform
Tang et al., 2010	Within-reconstruction PVC	Direct 4D reconstruction with the joint entropy
Le Pogam et al., 2011	Voxel-based PVC	3D voxel-wise mutual multiresolution algorithm
Wang and Fei, 2012	Voxel-based PVC	Voxel-based utilizing edge information on MR images
Sattarivand et al., 2012	Region-based PVC	Symmetric GTM method based on spillover between RSFs
Vunckx et al., 2012	Within-reconstruction PVC	Comparison among A-MAP,joint entropy and modified locally joint entropy
Coello et al., 2013	Voxel-based PVC	Hybrid voxel-region-based approach called LoReAn algorithm
Yan et al., 2015	Voxel-based PVC	MRI-guided filtering method
Tang and Rahmim, 2015	Within-reconstruction PVC	Wavelet-based JE MAP algorithm
Hutchcroft et al., 2016; Chen et al., 2019	Within-reconstruction PVC	Kernel method employing patch-based MR image features to form the matrix

*EM, expectation maximization; GTM, geometric transfer matrix; RSFs, regional spread functions; LoReAn, Local Regression Analysis; MAP, maximum a posteriori.*

### Region-Based PVC

The most common region-based PVC method using MRI anatomical data is geometric transfer matrix (GTM), which represents a linear transformation between the realistic regional activities and the raw data (Rousset et al., 1998). This preliminary GTM algorithm of MRI-based PVC attempted to correct regional activity in the comparatively thin gray matter affected by the nearby confounding tissues. The corrected value, or called recovery coefficient, obtained from MRI can be used to correct the mean raw value within the selected ROI (the target region) in 2D level. Then, this method was described in 3D mode with a sinogram implementation for PET striatal imaging using ^18^F-DOPA (Frouin et al., 2002). Besides regional evaluation, GTM algorithm also allows the activity correction for the entire brain simultaneously.

Compared to mis-registration, the errors in segmentation of the brain have been found to be of greater significance for the accuracy of PVC. In addition, the performance of segmentation performed on clinical MRI with lesions shows quite substantial differences from on MRI of normal subjects. It is suggested that segmentation using SPM is superior to HBSA or EMS in the GTM process for ^18^F-fluoro-deoxy-glucose (^18^F-FDG) and ^18^F-DOPA brain PET data (Zaidi et al., 2006). In the support of SPM and Freesurfer software, GTM method has been introduced into the PVC process for PDE10A enzyme and D2/3 receptors PET data (Fazio et al., 2017).

Recently, a novel analytically derived symmetric GTM (sGTM) method, depending on the spillover between regional spread functions rather than between regions, shows better noise characteristics and robustness compared with the conventional GTM method (Sattarivand et al., 2012). However, since these methods can only obtain the corrected regional mean activity, small lesions less than the size of the default ROI should remain under cover.

### Voxel-Based PVC

To further improve the entire brain PET image in voxel level after reconstruction, voxel-based PVC has been proposed as a feasible PVC method through enhancement or deconvolution guided by MRI anatomical information (Arakawa et al., 2017). Researchers use MRI anatomical data to create a brain tissue map by assigning the voxels value representing gray and white matters as 1, and CSF as 0 (Meltzer et al., 1990). This binary matrix is then convolved with the line spread function of PET to create a composite brain image assuming no background activity after co-registration. Correction of the PET tracer distribution from CSF, known as the MZ approach, is then performed in voxel level by dividing the actual PET image using the corresponding brain tissue map described above.

CSF and WM adjacent to GM also have different degree of ^18^F-FDG uptake, which of GM is about 4 times higher than WM (Kennedy et al., 1978). Different variants of these methods are referred to as two-(MZ), three-(MG), or four-compartment models (Meltzer et al., 1990, 1996; Müller-Gärtner et al., 1992). With these voxel-based strategies, a study coupling of diverse automated ROI placement and four alternative methods for PVC provides a software tool for flexible integrated analysis of brain PET/MRI data (Quarantelli et al., 2004). Regardless of introducing WM value calculation, two programs taking into account spillover effects between any feasible couple of ROIs, allows a recovery of actual GM activities with a higher degree of accuracy than others. An improved algorithm based on the theory of multiresolution analysis for images with different spatial resolutions can provide the possibility to acquire accurately corrected PET images without the definition of ROI (Boussion et al., 2006).

An alternative method based on mutual multiresolution analysis (MMA) algorithm restricted to the entire brain, adopts cerebral atlases as the reference of anatomical information, in order to minimize the mismatches between functional and anatomical data (Shidahara et al., 2009). However, this approach depends on the application of a 2D modeling, in which the correction is conducted slice by slice independently. Thus, artifacts may be introduced when significant correlation does not exist between anatomical and functional information. A novel model with 3D wavelet decomposition method has been designed to solve this issue, and proven in ^11^C-PIB and ^18^F-FDG PET imaging (Le Pogam et al., 2011; Grecchi et al., 2017). In addition, deconvolution-based PVC methods could generate voxel-based improved images without the requirement of MRI image segmentation (Wang and Fei, 2012). This simplified approach using edge information on MRI data rather than tissue classification information, can be particularly feasible for the PET images improvement in either separated or hybrid PET/MRI system.

Apart from GM, WM involvement has been known to exist in Alzheimer’s disease (AD) and several vascular diseases as well. Extensive examination of cerebral glucose metabolism or other pathophysiological states in WM using PET imaging, requires to develop PVC methods concerning the subcortical WM regions, in order to correct for the spill-out of activity from GM into WM (Coello et al., 2013). It consists of solving the convolution issue in voxel level by using Local Regression Analysis (LoReAn) with regional information derived from co-registered MRI images. Combined with voxel-wise MRI-guided filtering method and PVC, the optimization of PET images is more likely to be achieved (Yan et al., 2015). Definitely, most of these mentioned voxel-based PVC methods can be introduced into the process of PET data reconstruction.

### Within-Reconstruction PVC

Although promising improvements were noted in iterative deconvolution accompanied with regularization or denoising, it has been considered to be difficult for these post-reconstruction deconvolution-based methods to control redundant noise while obtaining sound resolution recovery (Le Pogam et al., 2011). Therefore, the within-reconstruction or reconstruction-based PVC method was developed to achieve more precise quantification of PET images (Reader et al., 2003).

Theoretically, the PET tracer distribution should be corresponding to the anatomical structure. In other words, all the voxels in the same tissue type are considered to have similar tracer uptake. Based on this assumption, the PVE on PET may induce the distribution discrepancy of raw data between PET and MRI. Therefore, a range of methods attempt to promote the PET image in line with the actual tracer distribution by incorporating priori anatomic models derived from MRI, within the reconstruction process rather than in a separate procedure after PET reconstruction. The commonly used algorithms involved in reconstruction are maximum *a posteriori* (MAP) or penalized likelihood formulation (Fessler et al., 1992; Baete et al., 2004). These within-reconstruction PVC methods have been also explored long before the advent of available hybrid PET/MRI system (Gindi et al., 1993; Sastry and Carson, 1997).

The original strategy of within-reconstruction PVC methods is based on anatomical boundary information derived from MRI, which defines corresponding boundaries of PET images (Fessler et al., 1992; Gindi et al., 1993; Ouyang et al., 1994; Su et al., 2017). In this form of PVC models, the iterative algorithm incorporates the anatomic data into the PET image reconstruction process within a Bayesian framework (Fessler et al., 1992). Besides, these anatomical data derived from MRI are considered as *a priori* information. The smoothing procedure is routinely used to reduce noise, but may further degrade the resolution in practice. Thus, the application of anatomical prior information allows for preventing the resolution degradation and preserving the inherent anatomic features of PET image (Gindi et al., 1993). A potential difficulty in this process is that some anatomical boundaries may not correspond to functional boundaries exactly. In other words, different functional distributions could exist in the identical anatomic region, whereas several functional distributions in different brain tissues might be quite similar. Therefore, a modified approach, in which only structural information having high joint probability with corresponding PET images are adopted, could provide further improvement in PET image quality (Ouyang et al., 1994).

Later, various methods were proposed by utilizing anatomical segmentation information, which facilitates a smooth tracer distribution within each anatomical region (Bowsher et al., 1996; Lipinski et al., 1997; Sastry and Carson, 1997; Rangarajan et al., 2000; Baete et al., 2004). The approximation of tissue model used in several post-reconstruction methods, can be included in the within-reconstruction PVC process as well (Bowsher et al., 1996; Baete et al., 2004). Similar to the post-reconstruction procedure, this algorithm was used to calculate the activity of each voxel within GM by using prior knowledge of activity distribution in the WM and CSF, on the basis of aligning the PET with anatomical information through state-of-the-art segmentation algorithm on high-resolution MRI (Baete et al., 2004). Based on the segmented MRI-guided tissue composition model, the activity is modeled as the sum of all contained tissue types in each individual voxel, and weighted by the composition fraction of each tissue type (Sastry and Carson, 1997). Then, the reconstruction algorithm formulates the corresponding activities of each tissue type at each voxel, within a Bayesian framework. To further handle the mismatch between anatomical and functional regions specifically, joint mixture model without requiring exactly homologous regions has been designed (Rangarajan et al., 2000).

Recently proposed PVC techniques use not only boundary or segmentation information, but also intensity signal to model the similarity between functional and anatomical images (Tang and Rahmim, 2009). This prior aims to generate homogeneous partitions in the PET image, in which the corresponding MRI also has an approximately homogeneous intensities distribution. Mutual information (MI) and its related joint entropy (JE) term have been introduced to create priors for PET image reconstruction without PVC (Somayajula et al., 2011). In the case with obvious disparities between anatomical and functional data, merely using MI tends to induce biased estimation in consideration of the marginal entropy term, whereas JE can serve as a more robust meter (Tang and Rahmim, 2009; van Golen et al., 2014).

The performance of the JE prior alone is not better than that of the other kinds of priors all the time (Vunckx et al., 2012). These within-reconstruction PVC measures only classify voxels based on the intensity values of MRI, while neglecting inherent spatial information of brain tissue. In a recent study, structural spatial information generated by using wavelet multi-resolution analysis is embedded in the JE between functional and anatomical image intensities (Tang and Rahmim, 2015). This modified MAP reconstruction algorithm involves derivatives of the subband JE measures in regard to the individual voxel intensities of PET image matrix. Compared to the intensity-only MAP algorithm, the wavelet-based JE-MAP algorithm yields comparable regional mean activities, and demonstrates robust performance in the clinical patient studies with PET and MRI data. Furthermore, significant enhancements in terms of SNR performance were also indicated, when performing directly parametric reconstruction of dynamic PET data by using the PET-MRI JE measure (Tang et al., 2010).

An alternative method for incorporating anatomical side information into the process of PET reconstruction based upon kernel methods has been proposed (Wang and Qi, 2015). This algorithm has obvious advantage of simplicity for the implementation using maximum likelihood expectation maximization (ML EM) reconstruction, and is initially focused on the reconstruction of dynamic PET data. Kernel based anatomically-aided reconstruction is derived from the patch-based MRI image features other than temporal features in dynamic PET reconstruction (Hutchcroft et al., 2016). It has been suggested that this Kernel method could be transplanted into the PET data optimization for simultaneous PET/MRI neuroimaging study (Chen et al., 2019).

### Clinical Application of MRI-Based PVC

The main target region for dopaminergic PET imaging is nigrostriatal system. Classical voxel-based PVC methods requiring tissue segmentation, assume regional homogeneous and known radioactivity levels for all regions except the target region for correction. These methods cannot solve the contamination between two or even more different GM structures. Since the activity level in striatum containing caudate and putamen is different from that in cortex, it is doubted that these voxel-based PVC methods are directly applied into striatal imaging with ^11^C-Raclopride or other dopaminergic tracers. Another potential limitation of voxel-based PVC is that the matrix scale for 3D implementation is substantially large. Because of large storage and calculative requirements, its clinical implementation subjects to the hardware configuration. It is a region-based method rather than a voxel-based method that avoids handling large matrices (Sattarivand et al., 2012). Region-based PVC methods still draw special attention in PET imaging, especially in striatal imaging (Smith et al., 2017).

Apart from the progressive atrophy of cerebral cortex, well-known pathological features of AD are amyloid plaque aggregation and tau pathology. The available tracers for the *in vivo* imaging of amyloid deposition includes ^11^C-PIB, ^18^F-florbetapir, ^18^F-florbetaben, and ^18^F-flutemetamol. The nonspecific tracer uptake in WM calls for correcting the contaminated signal of GM in amyloid PET imaging. MZ and MG voxel-based PVC methods have been utilized in ^18^F-flutemetamol and ^11^C-PIB PET imaging (Lowe et al., 2017). However, conventional anatomy-based PVC methods may result in severe biases in AD patients, since the degrees of cortical atrophy and Aβ deposition between various brain regions are heterogeneous. Region-based voxel-wise (RBV) correction, an improved PVC method, combines the benefits of GTM method and voxel-wise correction (Thomas et al., 2011). Homogeneity is assumed within a sub-region of cortex, but not necessarily within the entire GM or WM. Therefore, this RBV correction method is superior to conventional anatomy-based PVC methods in accounting for within-compartment variability.

Since each PVC method for PET may have very different property due to its given assumption and algorithms, the corrected PET images need careful interpretation. In a cross-sectional ^18^F-FDG PET study, the effects of aging on cerebral glucose uptake were analyzed with or without PVC (Greve et al., 2016). sGTM or GTM PVC methods are recommended in ROI analysis, while GTM-based RBV in voxel-wise analysis. In another recent study, five PVC methods, including MG, GTM, and other three methods, were applied to PET images using ^18^F-THK5351 or ^11^C-PIB (Shidahara et al., 2017). Different PVC methods result in different SUVRs within regions of high tracer uptake, and the degree of disparity between corrected and uncorrected images depends on PVC algorithm, type of tracer and subject condition. Though the similar mean values in each region of ^11^C-PIB PET images were observed between GTM and MG methods by using absolute quantitative analysis, further studies are still required to investigate the effect of voxel-wise PVC (Su et al., 2015b; Matsubara et al., 2016). Theoretically, MG method is not the best voxel-wise PVC method for amyloid PET studies, due to assuming homogeneous tracer uptake per tissue fractional volume in entire GM. Since the erroneous results stemming from this assumption are mostly predominant in voxels surrounded by regions with high ^11^C-PIB uptake, several improved voxel-wise analyses using RBV and Markov random field MRI-based PVC methods are preferable (Thomas et al., 2011; Bousse et al., 2012). However, the regional correspondence between amyloid PET and postmortem measures of amyloid load can not be obtained, whether or not applying PVC methods mentioned above (Minhas et al., 2018).

Another hallmark of AD, tau pathology, can be detected *in vivo* using PET tracer ^18^F-AV1451 or ^18^F-THK523. Unlike ^11^C-PIB, hippocampal ^18^F-THK523 retention is related to several cognitive parameters and the degree of hippocampal atrophy (Villemagne et al., 2014). Typical spatial distribution of aggregated tau in AD patients is substantial tracer retention in neocortical regions throughout temporal parietal and frontal cortex, and this distribution is remarkably different from that of young healthy adults or old adults. These results are similar after applying GTM PVC approach, except an obvious increase of choroid plexus retention adjacent to the hippocampus (Scholl et al., 2016; Baker et al., 2017).

Longitudinal clinical trials of AD progression and treatment response require semi-quantitative or quantitative approach for the analysis of brain amyloidosis. Besides the definition of reference regions, amyloid PET results are also affected by PVE in longitudinal studies, especially in patients with obvious atrophy (Brendel et al., 2015). The annual rate of amyloid deposition detected using ^11^C-PIB is 3.4 times greater by GTM approach and 1.59 times greater with MZ approach in comparison to the degree without any PVC (Su et al., 2015b). The impact of modified voxel-based MG PVC method on ^18^F-florbetaben PET images is considered to be associated with the degree of atrophy (Rullmann et al., 2016). Therefore, this PVC method increases the capability to discriminate between AD patients and healthy subjects, and the potential to investigate dynamic changes in brain amyloid deposition over time using ^18^F-florbetaben PET imaging. Based on the comparative analysis of 1024 software pipelines with diverse combinations of methodological choices, the optimal longitudinal measure of amyloid deposition using ^11^C-PIB should be the combination of reference region including voxels in the WM and the whole cerebellum with two-compartment MZ PVC approach (Schwarz et al., 2017). Measurements using GTM PVC approach have significantly worse within-subject variability than those using MZ, MG, or no PVC (Schwarz et al., 2019). Consequently, in longitudinal amyloid PET, GTM PVC method is considered to be less suitable than other traditional approaches when investigating within-person alteration over time. Compared to amyloid PET study, rare evaluation of PVC approaches has been carried out in longitudinal tau PET study. Mean rates of within-subject variance over time using MZ PVC are slightly more obvious than those without PVC in longitudinal ^18^F-AV1451 PET study (Jack et al., 2018). It remains an open question what PVC algorithm is the optimal approach for serial tau PET imaging.

## MRI-Based Quantitative Analysis

PET using ^15^O-tracers are considered to be the golden standard for measuring cerebral blood flow (CBF), cerebral blood volume (CBV), oxygen extraction fraction (OEF), and cerebral metabolic rate of oxygen (CMRO_2_). The quantitative analysis of these dynamic PET data relies upon an invasive arterial blood sampling procedure to acquire AIF, which is mandatory for kinetic models to calculate the rate constants between different compartments. The AIF refers to the time activity curve (TAC) of injected tracer in arterial blood plasma delivered to the target tissue. This laborious invasive procedure not only is vulnerable to the measurement error, but also may induce potential risk of complications. In addition, due to the strong magnetic field in and around hybrid PET/MRI scanner, arterial sampling becomes more challenging and requires additional specialized equipment. Therefore, it is extremely valuable to develop a robust AIF estimation technique to quantify brain hemodynamic parameters, without requiring invasive arterial blood sampling.

IDIF method obtained from PET data itself, can serve as a potential alternative estimation of AIF. Blood pool, ventricles of the heart or major vessels, has been taken to measure the IDIF. Several studies define the ROI of blood pool as reference region directly on the PET images alone. To reduce the spillover and PVE, the approach of ROI definition can be combined with anatomical information from MRI, other than with PET data only. Initially, ROIs on MRI images are drawn to delineate the internal carotid arteries. Depending on precise co-registration and segmentation, ROIs derived from MRI are transferred into the PET images of the same subject. Then, the TAC can be calculated for each ROI to estimate the blood input function based on MRMC and PVC. This method by extracting IDIF rather than measuring arterial blood-sampling has been validated in ^18^F-FDG studies (Chen et al., 1998). The combination of PET information with MRI segmented regions, especially by segmenting the left and right carotid arteries separately, demonstrates an improvement over regions based solely on MRI or PET alone (Fung et al., 2009). Apart from co-registration and segmentation, the MRI images can be also used to delineate the centerline of each carotid. High resolution PET, guided by MRI-based IDIF using carotid centerline method and a fixed recovery coefficient, allows for the noninvasive CBF measurements (Fung and Carson, 2013).

To achieve more reliable tissue segmentation, several 3D approaches with higher spatial resolution have been proposed for determination of IDIF (Caldeira et al., 2015). Magnetic resonance angiography (MRA) is a group of MRI techniques to image blood vessels including the arteries inside the brain and neck. Owing to the short echo time and flow compensation, time-of-flight (TOF) MRA makes the flowing blood brighter than other static tissue. Via precise location of the carotid artery identified in MRA, a technique for IDIF measurement based on co-registered MRA images has been developed and validated in the quantitative analysis of ^15^O-H_2_O PET study (Su et al., 2013; Okazawa et al., 2018). In addition to the feasibility for the reproducible CBF estimation without arterial cannulization, good agreement is also observed between MRA-based IDIF and conventional arterial blood sampling. With the support of co-registered TOF-MRA images, IDIF extracted from ^11^C-PIB PET imaging has been also applied for the amyloid imaging quantification (Su et al., 2015a). However, this technique is supposed to be sensitive to co-registration errors. Since the motion of head or neck can change the carotid position and shape, the co-registration of sequentially acquired anatomical and functional modalities may be difficult.

This TOF MRA-based IDIF method could be further implemented into hybrid PET/MRI system, so as to quantify the OEF and CBV more accurately (Su et al., 2017). Furthermore, after optimizing the dose of ^15^O-H_2_O in hybrid device, a short time-frame PET angiogram during arterial phase can be reconstructed with sufficient counts (Khalighi et al., 2018). This PET angiogram data has the potential to measure the extent of spill-over, and the IDIF guided by the true arterial volume based on TOF MRA. Since manual operation should be minimized in IDIF process, fully automated generation of IDIF using simultaneous PET/MRI without arterial catheterization is ideal in clinical practice (Jochimsen et al., 2016).

Hybrid MRI-PET device offers new insights into different brain status by simultaneously assessing cerebral oxygen consumption, metabolism and perfusion. The superiority for the combination of functional measurements from both modalities is considered to be far beyond anatomical co-registration only. AIF obtained from the Gd-DTPA in MRI can be converted into ^18^F-FDG AIF in PET, while metabolic rates of glucose calculated with AIFs derived from two modalities also found no statistical difference (Poulin et al., 2013). This dynamic contrast-enhanced MRI data provides high temporal resolution, which is the primary prerequisite for the accurate pharmacokinetic parameters estimation. A limitation of this conversion method is the need of at least one accurate blood sampling. Therefore, an upgraded conversion method using AIF derived from an reference region in MRI with Gd-DTPA has been proposed (Poulin et al., 2015). Another possible strategy is the automatic detection for the first pass bolus of Gd-DTPA. Automatically detecting blood signal enables the direct IDIF extraction to obviate the procedure of reference tissue delineation (Evans et al., 2013).

Phase-contrast (PC) MRI utilizing phase differences to distinguish blood signal from other static tissues, is an established imaging technique for estimation of global CBF (Vestergaard et al., 2017). Global CBF detected by PC MRI may be applied as a reference region for quantitative analysis of PET data to avoid the need of blood sampling. Since ^15^O-H_2_O PET and PC MRI are acquired simultaneously in hybrid system, it adds no additional scanning time for the accurate measurements of CBF (Ssali et al., 2018).

## Future Perspective

At present, there are abundant available fusion software for fusing PET data with separately acquired MRI images satisfactorily (Ding et al., 2018). However, only hybrid PET/MRI system can provide the real time matched functional information representing disease states, which may undergo changes between the two separated imaging studies. Since various studies apply different MC procedures and analysis methods, no clear consensus of its clinical value has been achieved. The clinical application of MRMC is only just beginning, and its technical evidence for brain PET study is encouraging.

**FIGURE 2:**
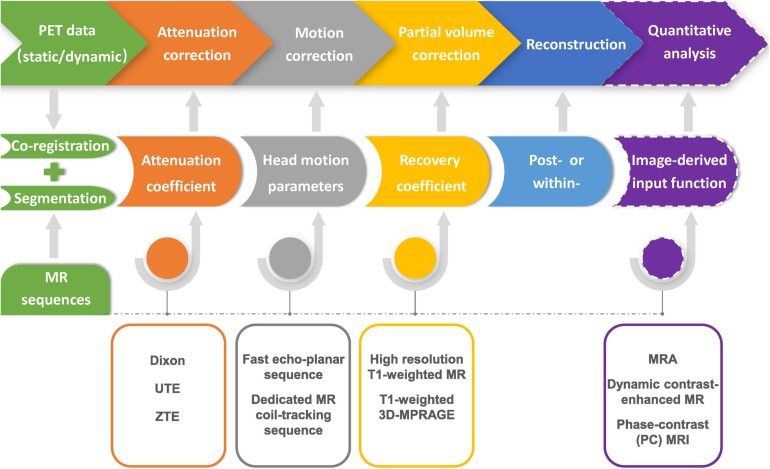
Flow chart of MR-based PET data optimization and reconstruction. Corrections for photon attenuation, head motion and partial volume effect of PET data can be achieved by using specific MR sequences, and conducted in the format of either within- or post-reconstruction. Precise image co-registration and segmentation are the prerequisite for various MR-based PET data optimization procedures. MRI-based image-derived input function method could be further applied in the quantitative analysis of dynamic PET imaging.

PET/MRI technique is an quickly changing field, in which lots of methodologies are maturing (Cabello and Ziegler, 2018). A workflow of MRI-based PET optimization and reconstruction is illustrated in Figure 2. Despite the obvious merits and bright prospects of hybrid PET/MRI device, there are still several points in favor of sequential PET/MRI acquisitions. First of all, the two devices could be also operated separately on occasion, which significantly reduces the redundant costs. Secondly, pharmacokinetic time scales of many tracers are not exactly matched between PET and MRI examinations. The most commonly used PET agent, ^18^F-FDG, requires an interval of uptake time during which MRI data can be obtained in separated device. Last but not least, hybrid PET/MRI system will still face more technologic challenges compared with those of sequential PET/MRI device in the near future (Wehrl et al., 2015).

Attenuation correction (AC) methods for PET require information of the spatial parameters of tissue attenuation coefficients, which is generally represented in the form of attenuation map. CT-based AC for PET data has been considered as the “golden standard” in clinical imaging. MRI, however, cannot provide information on electron density, which is critical for the process of PET AC. By far, the MRI-based AC for PET data is still one of the major challenges in obtaining accurate PET quantification in hybrid PET/MRI imaging system (Chen and An, 2017). The MRI-based AC strategies that have been extensively pursued especially for brain imaging, can be divided into two classes: atlas-based approaches and segmentation-based approaches. Atlas-based AC methods allow the accurate attenuation maps for PET brain imaging, especially for patients without abnormal anatomy. Complex computations in this approach are time consuming, which handicaps its widely clinical application. Compared with atlas-based AC methods, segmentation-based AC methods need shorter computation time and can account for anatomic variation. To further distinguish bony structure from air accurately, several novel methods based on machine learning have been developed to effectively capture the relationship between the CT and MRI images and derive the attenuation map (Lei et al., 2018; Yang et al., 2019). Since database of pediatric age-matched healthy subjects is difficult to acquire, MRI-based AC methods targeted toward this age group are scarce. With the development of machine learning, deep learning in particular, this issue may be solved properly. In view of the multiparametric strengths of MRI, generating attenuation maps using multiple MRI inputs would be another promising research direction (Gong et al., 2018).

The premise for the MRI-based MC and PVC is the similar distribution in specific tissue between these two modalities. However, it is possible that the distribution pattern of functional information provided by PET is not in accordance with the MRI-based structural information all the time. Therefore, the rationality of using MRI to guide the PET data process may be doubted. This issue calls for a robust approach to evaluate the accuracy of MRI-based correction methods. Though there are various phantoms available for different purposes, such as calibrations of physical performance, dose algorithm accuracy, geometrical accuracy and image quality, the complex structure and geometry of brain cannot be fully imitated (Beichel et al., 2017). Therefore, the piecewise-uniform distributions of these phantoms may mislead the analysis of MRI-based correction methods to a certain extent. In order to overcome these problems, the emerging 3D printing technology has the potential to offer more versatile and accurate solutions (Filippou and Tsoumpas, 2018).

The majority of the MC procedures are complex, multi-step and require simultaneous acquisition of PET and MRI data. Accurate path of head motion measured by simultaneous MRI could generate precise reference trajectory for the PET MC. However, continuous monitor with such manner blocks the diversified utility of MRI for other informative sequences. In addition, MRMC is generally related to prominent costs, and it is unclear whether the MRMC-related clinical benefits outweigh it. On the other hand, reducing demands on MRI navigator during the course of PET imaging can decrease the MRMC-related costs. Since fast motions cannot be accurately detected with excessively low sampling rate of specified MRI navigator sequence, a degree of compromise between MRMC-related costs and sampling quality should be achieved. Several researchers suggested that direct reconstruction using event-by-event motion correction for dynamic brain PET is achievable and robust (Germino et al., 2017; Jiao et al., 2017). On the other hand, accurate MC may be achieved at a cost of resolution loss to some extent (Reilhac et al., 2018). These issue may also be solved by the combination of MRI navigator sequence and optical motion tracking system, which complement each other’s advantages. In the near future, researches focused on how MRMC influences the particular tasks for brain PET imaging are extremely expected.

In the MRI-based PVC process, both the co-registration of brain PET data and anatomical information derived from MRI and the segmentation of brain regions maintaining a relatively uniform distribution of tracer uptake are the two critical beforehand steps. The effect of PVC would be vulnerable to the errors occurring in the procedure of either co-registration or segmentation. Besides, this detrimental influence on the accuracy of PVC algorithms not only relates to the region-based approach, but also the voxel-based one. A robust image segmentation procedure, other than the conventional ROI analysis, is in desperate need for the process and analysis of images. Predictably, joint segmentation methods integrating anatomical information from MRI with functional data from PET would facilitate the interpenetration of two images within uniform platform, and the more precise delineation of brain.

Though MRI-based IDIF is an attractive noninvasive alternative to arterial blood sampling, it is also a very challenging field associated with diverse problems impeding its clinical utilization. Due to the absence of large blood pools in brain, IDIF can only depend on intracranial blood vessels. Most algorithms for PET and MRI co-registration are based on the rigid brain structures rather than carotids. In addition, the carotid is a relatively small and elastic tissue. The position change of head or neck can elongate and bend the carotid. As a result, a precise brain co-registration cannot promise a good co-registrations of the carotid arteries. Since the degree of mismatch between the PET and MRI images for one carotid may be different from the contralateral one, it is also necessary to co-register the left and right carotids independently by complicated co-registration algorithms in separated PET/MRI device. Definitely, the hybrid PET/MRI device could serve as a perfect solution to minimize this problem.

For pediatric patients, the superiority of PET/MRI is associated with the reduction of radiation exposure and the shortening of anesthesia or sedation duration. Nevertheless, the brain glucose metabolism and other functional states undergo dynamic change throughout the whole course of brain development. It must be mentioned that the regional glucose utilization of different brain regions does not change in a parallel pattern with age. Thus, the variable brain sizes and metabolism patterns of pediatric patients may induce dramatic artifacts in these MRI-based correction procedures. Extensive efforts are still required to refine the application of these methods in this special and considerable population.

As discussed above, MRI images acquired either sequentially or simultaneously can be applied to improve the PET data quality. On the other side, optimized PET data could be also used to verify a variety of functional MRI techniques. This cross-calibration method allows the validation of MRI-based functional parameters, including cerebral perfusion, neuronal activation, brain rest state, brain connectivity and so on. Taking the cerebral perfusion for example, discrepancies between cerebral perfusion derived from MRI and CBF evaluated by ^15^O-H_2_O PET imaging may be obvious. The utilization of simultaneous PET/MRI device affords the opportunity for cross-validation of different techniques, and the further definition of techniques confounds in this basic physiological measurement. In addition, brain functional connectivity between different regions can be measured by fMRI procedures, in the absence of specific neurotransmitters information of these connected areas. Undoubtedly, the integral map of brain functional connectivity requires intensive collaboration between PET and MRI. Higher level of information synthesis from both PET and MRI to acquire a brand-new parameter of brain function is an attracting field, which is still relatively unexplored.

## Author Contributions

YZ and XZ conceived the review and reviewed the literature. YZ wrote the manuscript. XZ revised the manuscript. Both authors finalized the manuscript and approved it for publication.

## Conflict of Interest Statement

The authors declare that the research was conducted in the absence of any commercial or financial relationships that could be construed as a potential conflict of interest.

## References

[B1] Aiello M. Cavaliere C. Salvatore M. (2016). Hybrid PET/MR imaging and brain connectivity. *Front. Neurosci.* 10:64. 10.3389/fnins.2016.00064 26973446 PMC4771762

[B2] Aksoy M. Forman C. Straka M. Skare S. Holdsworth S. Hornegger J. (2011). Real-time optical motion correction for diffusion tensor imaging. *Magn. Reson. Med.* 66 366–378. 10.1002/mrm.22787 21432898 PMC3139706

[B3] Arakawa R. Stenkrona P. Takano A. Nag S. Maior R. S. Halldin C. (2017). Test-retest reproducibility of [^11^C]-L-deprenyl-D2 binding to MAO-B in the human brain. *EJNMMI Res.* 7:54. 10.1186/s13550-017-0301-4 28634836 PMC5478550

[B4] Ardekani B. A. Braun M. Hutton B. F. Kanno I. Iida H. (1996). Minimum cross-entropy reconstruction of PET images using prior anatomical information. *Phys. Med. Biol.* 41 2497–2517. 10.1088/0031-9155/41/11/018 8938041

[B5] Baete K. Nuyts J. Van Laere K. Van Paesschen W. Ceyssens S. De Ceuninck L. (2004). Evaluation of anatomy based reconstruction for partial volume correction in brain FDG-PET. *Neuroimage* 23 305–317. 10.1016/j.neuroimage.2004.04.041 15325378

[B6] Baker S. L. Maass A. Jagust W. J. (2017). Considerations and code for partial volume correcting [^18^F]-AV-1451 tau PET data. *Data Brief* 15 648–657. 10.1016/j.dib.2017.10.024 29124088 PMC5671473

[B7] Beichel R. R. Smith B. J. Bauer C. Ulrich E. J. Ahmadvand P. Budzevich M. M. (2017). Multi-site quality and variability analysis of 3D FDG PET segmentations based on phantom and clinical image data. *Med. Phys.* 44 479–496. 10.1002/mp.12041 28205306 PMC5834232

[B8] Blume M. Martinez-Möller A. Keil A. Navab N. Rafecas M. (2010). Joint reconstruction of image and motion in gated positron emission tomography. *IEEE Trans. Med. Imaging* 29 1892–1906. 10.1109/tmi.2010.2053212 20562034

[B9] Bousse A. Bertolli O. Atkinson D. Arridge S. Ourselin S. Hutton B. F. (2016). Maximum-likelihood joint image reconstruction/motion estimation in attenuation-corrected respiratory gated PET/CT using a single attenuation map. *IEEE Trans. Med. Imaging* 35 217–228. 10.1109/tmi.2015.2464156 26259017

[B10] Bousse A. Pedemonte S. Thomas B. A. Erlandsson K. Ourselin S. Arridge S. (2012). Markov random field and gaussian mixture for segmented MRI-based partial volume correction in PET. *Phys. Med. Biol.* 57 6681–6705. 10.1088/0031-9155/57/20/6681 23023073

[B11] Boussion N. Hatt M. Lamare F. Bizais Y. Turzo A. Cheze-Le Rest C. (2006). A multiresolution image based approach for correction of partial volume effects in emission tomography. *Phys. Med. Biol.* 51 1857–1876. 10.1088/0031-9155/51/7/016 16552110

[B12] Bowsher J. E. Johnson V. E. Turkington T. G. Jaszczak R. J. Floyd C. R. Coleman R. E. (1996). Bayesian reconstruction and use of anatomical a priori information for emission tomography. *IEEE Trans. Med. Imaging.* 15 673–686. 10.1109/42.538945 18215949

[B13] Brendel M. Hogenauer M. Delker A. Sauerbeck J. Bartenstein P. Seibyl J. (2015). Improved longitudinal [^18^F]-AV45 amyloid PET by white matter reference and VOI-based partial volume effect correction. *Neuroimage* 108 450–459. 10.1016/j.neuroimage.2014.11.055 25482269

[B14] Cabello J. Ziegler S. I. (2018). Advances in PET/MR instrumentation and image reconstruction. *Br. J. Radiol.* 91:20160363. 10.1259/bjr.20160363 27376170 PMC5966194

[B15] Caldeira L. Yun S. D. da Silva N. Filss C. Scheins J. Telmann L. (2015). Simultaneous acquisition of dynamic PET-MRI: arterial input function using DSC-MRI and [18F]-FET. *EJNMMI Phys.* 2:A70 (Suppl. 1). 10.1186/2197-7364-2-s1-a70 26956331 PMC4798684

[B16] Cal-Gonzalez J. Perez-Liva M. Herraiz J. L. Vaquero J. J. Desco M. Udias J. M. (2015). Tissue-dependent and spatially-variant positron range correction in 3D PET. *IEEE Trans. Med. Imaging* 34 2394–2403. 10.1109/tmi.2015.2436711 26011878

[B17] Catana C. Benner T. van der Kouwe A. Byars L. Hamm M. Chonde D. B. (2011). MRI-Assisted PET motion correction for neurologic studies in an integrated MR-PET scanner. *J. Nucl. Med.* 52 154–161. 10.2967/jnumed.110.079343 21189415 PMC3125596

[B18] Chen K. Bandy D. Reiman E. Huang S. C. Lawson M. Feng D. (1998). Noninvasive quantification of the cerebral metabolic rate for glucose using positron emission tomography, 18F-fluoro-2-deoxyglucose, the patlak method, and an image-derived input function. *J. Cereb. Blood Flow Metab.* 18 716–723. 10.1097/00004647-199807000-00002 9663501

[B19] Chen K. T. Salcedo S. Chonde D. B. Izquierdo-Garcia D. Levine M. A. Price J. C. (2018). MR-assisted PET motion correction in simultaneous PET/MRI studies of dementia subjects. *J. Magn. Reson. Imaging.* 48 1288–1296. 10.1002/jmri.26000 29517819 PMC6129224

[B20] Chen K. T. Salcedo S. Gong K. Chonde D. B. Izquierdo-Garcia D. Drzezga A. E. (2019). An efficient approach to perform MR-assisted PET data optimization in simultaneous PET/MR neuroimaging studies. *J. Nucl. Med.* 60 272–278. 10.2967/jnumed.117.207142 29934405 PMC8833859

[B21] Chen Y. An H. (2017). Attenuation correction of PET/MR imaging. *Magn. Reson. Imaging Clin. N. Am.* 25 245–255. 10.1016/j.mric.2016.12.001 28390526 PMC5385843

[B22] Chun S. Y. Reese T. G. Ouyang J. Guerin B. Catana C. Zhu X. (2012). MRI-Based nonrigid motion correction in simultaneous PET/MRI. *J. Nucl. Med.* 53 1284–1291. 10.2967/jnumed.111.092353 22743250 PMC4077320

[B23] Coello C. Willoch F. Selnes P. Gjerstad L. Fladby T. Skretting A. (2013). Correction of partial volume effect in 18F-FDG PET brain studies using coregistered MR volumes: voxel based analysis of tracer uptake in the white matter. *NeuroImage* 72 183–192. 10.1016/j.neuroimage.2013.01.043 23370062

[B24] Ding Y. Zhu Y. Jiang B. Zhou Y. Jin B. Hou H. (2018). (18)F-FDG PET and high-resolution MRI co-registration for pre-surgical evaluation of patients with conventional MRI-negative refractory extra-temporal lobe epilepsy. *Eur. J. Nucl. Med. Mol. Imaging* 45 1567–1572. 10.1007/s00259-018-4017-0 29671038

[B25] Erlandsson K. Buvat I. Pretorius P. H. Thomas B. A. Hutton B. F. (2012). A review of partial volume correction techniques for emission tomography and their applications in neurology, cardiology and oncology. *Phys. Med. Biol.* 57 R119–R159. 10.1088/0031-9155/57/21/r119 23073343

[B26] Erlandsson K. Dickson J. Arridge S. Atkinson D. Ourselin S. Hutton B. F. (2016). MR imaging-guided partial volume correction of PET data in PET/MR imaging. *PET Clin.* 11 161–177. 10.1016/j.cpet.2015.09.002 26952729

[B27] Evans E. Sawiak S. J. Adrian Carpenter T. (2013). MRI-derived arterial input functions for PET kinetic modelling in rats. *Nucl. Instrum. and Methods Phys. Res. A.* 702 126–128. 10.1016/j.nima.2012.08.081

[B28] Fazio P. Schain M. Mrzljak L. Amini N. Nag S. Al-Tawil N. (2017). Patterns of age related changes for phosphodiesterase type-10A in comparison with dopamine D2/3 receptors and sub-cortical volumes in the human basal ganglia: a PET study with (18)F-MNI-659 and (11)C-raclopride with correction for partial volume effect. *Neuroimage* 152 330–339. 10.1016/j.neuroimage.2017.02.047 28254508

[B29] Fessler J. A. Clinthorne N. H. Rogers W. L. (1992). Regularized emission image reconstruction using imperfect side information. *IEEE Trans. Nucl. Sci.* 39 1464–1471. 10.1109/23.173225

[B30] Filippou V. Tsoumpas C. (2018). Recent advances on the development of phantoms using 3D printing for imaging with CT, MRI, PET, SPECT and ultrasound. *Med. Phys.* 45 e740–e760. 10.1002/mp.13058 29933508 PMC6849595

[B31] Frouin V. Comtat C. Reilhac A. Grégoire M. (2002). Correction of partial-volume effect for PET striatal imaging: fast implementation and study of robustness. *J. Nucl. Med.* 43 1715–1726. 12468524

[B32] Fung E. K. Carson R. E. (2013). Cerebral blood flow with [15O]water pet studies using an image-derived input function and MR-defined carotid centerlines. *Phys. Med. Biol.* 58 1903–1923. 10.1088/0031-9155/58/6/1903 23442733 PMC3626495

[B33] Fung E. K. Planeta-Wilson B. Mulnix T. Carson R. E. (2009). “A multimodal approach to image-derived input functions for brain PET,” in *Proceedings of the 2009 IEEE Nuclear Science Symposium Conference Record (NSS/MIC)* (Orlando, FL: IEEE), 2710–2714. 10.1109/NSSMIC.2009.5401977 PMC2895272 20607124

[B34] Fürst S. Grimm R. Hong I. Souvatzoglou M. Casey M. E. Schwaiger M. (2015). Motion correction strategies for integrated PET/MR. *J. Nucl. Med.* 56 261–269. 10.2967/jnumed.114.146787 25572092

[B35] Gambhir S. S. (2002). Molecular imaging of cancer with positron emission tomography. *Nat. Rev. Cancer* 2 683–693. 10.1038/nrc882 12209157

[B36] Germino M. Gallezot J. D. Yan J. Carson R. E. (2017). Direct reconstruction of parametric images for brain PET with event-by-event motion correction: evaluation in two tracers across count levels. *Phys. Med. Biol.* 62 5344–5364. 10.1088/1361-6560/aa731f 28504644 PMC5783541

[B37] Gindi G. Lee M. Rangarajan A. Zubal I. (1993). Bayesian reconstruction of functional images using anatomical information as priors. *IEEE Trans. Med. Imaging* 12 670–680. 10.1109/42.251117 18218461

[B38] Gong K. Yang J. Kim K. El Fakhri G. Seo Y. Li Q. (2018). Attenuation correction for brain PET imaging using deep neural network based on dixon and ZTE MR images. *Phys. Med. Biol.* 63:125011. 10.1088/1361-6560/aac763 29790857 PMC6031313

[B39] Grecchi E. Veronese M. Bodini B. Garcia-Lorenzo D. Battaglini M. Stankoff B. (2017). Multimodal partial volume correction: application to [^11^C]PIB PET/MRI myelin imaging in multiple sclerosis. *J. Cereb. Blood Flow Metab.* 37 3803–3817. 10.1177/0271678x17712183 28569617 PMC5718330

[B40] Greve D. N. Salat D. H. Bowen S. L. Izquierdo-Garcia D. Schultz A. P. Catana C. (2016). Different partial volume correction methods lead to different conclusions: an (18)F-FDG-PET study of aging. *Neuroimage* 132 334–343. 10.1016/j.neuroimage.2016.02.042 26915497 PMC4851886

[B41] Gumus K. Keating B. White N. Andrews-Shigaki B. Armstrong B. Maclaren J. (2015). Comparison of optical and MR-based tracking. *Magn. Reson. Med.* 74 894–902. 10.1002/mrm.25472 25257096 PMC4372516

[B42] Gutierrez D. Montandon M. Assal F. Allaoua M. Ratib O. Lövblad K. (2012). Anatomically guided voxel-based partial volume effect correction in brain PET: impact of MRI segmentation. *Comput. Med. Imaging Graph* 36 610–619. 10.1016/j.compmedimag.2012.09.001 23046730

[B43] Herzog H. Tellmann L. Fulton R. Stangier I. Rota K. E. Bente K. (2005). Motion artifact reduction on parametric PET images of neuroreceptor binding. *J. Nucl. Med.* 46 1059–1065. 15937320

[B44] Holman B. F. Cuplov V. Millner L. Hutton B. F. Maher T. M. Groves A. M. (2015). Improved correction for the tissue fraction effect in lung PET/CT imaging. *Phys. Med. Biol.* 60 7387–7402. 10.1088/0031-9155/60/18/7387 26350580

[B45] Huang C. Ackerman J. L. Petibon Y. Brady T. J. El Fakhri G. Ouyang J. (2014a). MR-based motion correction for PET imaging using wired active MR microcoils in simultaneous PET-MR: phantom study. *Med. Phys.* 41:041910. 10.1118/1.4868457 24694141 PMC3978416

[B46] Huang C. Ackerman J. L. Petibon Y. Normandin M. D. Brady T. J. El Fakhri G. (2014b). Motion compensation for brain PET imaging using wireless MR active markers in simultaneous PET–MR: phantom and non-human primate studies. *NeuroImage* 91 129–137. 10.1016/j.neuroimage.2013.12.061 24418501 PMC3965607

[B47] Hutchcroft W. Wang G. Chen K. T. Catana C. Qi J. (2016). Anatomically-aided PET reconstruction using the kernel method. *Phys. Med. Biol.* 61 6668–6683. 10.1088/0031-9155/61/18/6668 27541810 PMC5095621

[B48] Ikari Y. Nishio T. Makishi Y. Miya Y. Ito K. Koeppe R. A. (2012). Head motion evaluation and correction for PET scans with 18F-FDG in the Japanese alzheimer’s disease neuroimaging initiative (J-ADNI) multi-center study. *Ann. Nucl. Med.* 26 535–544. 10.1007/s12149-012-0605-4 22763629

[B49] Jack C. R. Jr. Wiste H. J. Schwarz C. G. Lowe V. J. Senjem M. L. Vemuri P. (2018). Longitudinal tau PET in ageing and alzheimer’s disease. *Brain* 141 1517–1528. 10.1093/brain/awy059 29538647 PMC5917767

[B50] Jiao J. Bousse A. Thielemans K. Burgos N. Weston P. S. Schott J. M. (2017). Direct parametric reconstruction with joint motion estimation/correction for dynamic brain PET data. *IEEE Trans. Med. Imaging* 36 203–213. 10.1109/tmi.2016.2594150 27576243

[B51] Jin X. Mulnix T. Gallezot J. D. Carson R. E. (2013). Evaluation of motion correction methods in human brain PET imaging–a simulation study based on human motion data. *Med. Phys.* 40:102503. 10.1118/1.4819820 24089924 PMC3785538

[B52] Jochimsen T. H. Zeisig V. Schulz J. Werner P. Patt M. Patt J. (2016). Fully automated calculation of image-derived input function in simultaneous PET/MRI in a sheep model. *EJNMMI. Phys.* 3:2. 10.1186/s40658-016-0139-2 26872658 PMC4752572

[B53] Jones T. Rabiner E. A. (2012). The development, past achievements, and future directions of brain PET. *J. Cereb. Blood Flow Metab.* 32 1426–1454. 10.1038/jcbfm.2012.20 22434067 PMC3390812

[B54] Keller S. Hansen C. Hansen C. Andersen F. Ladefoged C. Svarer C. (2015). Motion correction in simultaneous PET/MR brain imaging using sparsely sampled MR navigators: a clinically feasible tool. *EJNMMI Phys.* 2:14. 10.1186/s40658-015-0118-z 26501815 PMC4538713

[B55] Kennedy C. Sakurada O. Shinohara M. Jehle J. Sokoloff L. (1978). Local cerebral glucose utilization in the normal conscious macaque monkey. *Ann. Neurol.* 4 293–301. 10.1002/ana.410040402 103488

[B56] Khalighi M. M. Deller T. W. Fan A. P. Gulaka P. K. Shen B. Singh P. (2018). Image-derived input function estimation on a TOF-enabled PET/MR for cerebral blood flow mapping. *J. Cereb. Blood Flow Metab.* 38 126–135. 10.1177/0271678x17691784 28155582 PMC5757438

[B57] Kustner T. Schwartz M. Martirosian P. Gatidis S. Seith F. Gilliam C. (2017). MR-based respiratory and cardiac motion correction for PET imaging. *Med. Image Anal.* 42 129–144. 10.1016/j.media.2017.08.002 28800546

[B58] Kyme A. Z. Se S. Meikle S. R. Fulton R. R. (2018). Markerless motion estimation for motion-compensated clinical brain imaging. *Phys. Med. Biol.* 63:105018. 10.1088/1361-6560/aabd48 29637899

[B59] Lalush D. S. (2017). Magnetic resonance-derived improvements in PET imaging. *Magn. Reson. Imaging Clin. N. Am.* 25 257–272. 10.1016/j.mric.2016.12.002 28390527

[B60] Le Pogam A. Hatt M. Descourt P. Boussion N. Tsoumpas C. Turkheimer F. E. (2011). Evaluation of a 3D local multiresolution algorithm for the correction of partial volume effects in positron emission tomography. *Med. Phys.* 38 4920–4933. 10.1118/1.3608907 21978037 PMC3485215

[B61] Lei Y. Shu H. K. Tian S. Wang T. Liu T. Mao H. (2018). Pseudo CT estimation using patch-based joint dictionary learning. *Conf. Proc. IEEE Eng. Med. Biol. Soc.* 2018 5150–5153. 10.1109/embc.2018.8513475 30441499

[B62] Lipinski B. Herzog H. Kops E. R. Oberschelp W. Muller-Gartner H. W. (1997). Expectation maximization reconstruction of positron emission tomography images using anatomical magnetic resonance information. *IEEE Trans. Med. Imaging* 16 129–136. 10.1109/42.563658 9101322

[B63] Lowe V. J. Lundt E. Knopman D. Senjem M. L. Gunter J. L. Schwarz C. G. (2017). Comparison of [^18^F]flutemetamol and [^11^C]pittsburgh compound-B in cognitively normal young, cognitively normal elderly, and alzheimer’s disease dementia individuals. *Neuroimage Clin.* 16 295–302. 10.1016/j.nicl.2017.08.011 28856092 PMC5565786

[B64] Manber R. Thielemans K. Hutton B. F. Barnes A. Ourselin S. Arridge S. (2015). Practical PET respiratory motion correction in clinical PET/MR. *J. Nucl. Med.* 56 890–896. 10.2967/jnumed.114.151779 25952740

[B65] Marner L. Henriksen O. M. Lundemann M. Larsen V. A. Law I. (2017). Clinical PET/MRI in neurooncology: opportunities and challenges from a single-institution perspective. *Clin. Transl. Imaging* 5 135–149. 10.1007/s40336-016-0213-8 28936429 PMC5581366

[B66] Matsubara K. Ibaraki M. Shimada H. Ikoma Y. Suhara T. Kinoshita T. (2016). Impact of spillover from white matter by partial volume effect on quantification of amyloid deposition with [^11^C]PiB PET. *Neuroimage* 143 316–324. 10.1016/j.neuroimage.2016.09.028 27639351

[B67] Matsunaga K. Yanagawa M. Otsuka T. Hirata H. Kijima T. Kumanogoh A. (2017). Quantitative pulmonary blood flow measurement using ^15^O-H_2_O PET with and without tissue fraction correction: a comparison study. *EJNMMI Res.* 7:102. 10.1186/s13550-017-0350-8 29274016 PMC5741573

[B68] Meechai T. Tepmongkol S. Pluempitiwiriyawej C. (2015). Partial-volume effect correction in positron emission tomography brain scan image using super-resolution image reconstruction. *Br. J. Radiol.* 88:20140119. 10.1259/bjr.20140119 25492553 PMC4614236

[B69] Meltzer C. C. Kinahan P. E. Greer P. J. Nichols T. E. Comtat C. Cantwell M. N. (1999). Comparative evaluation of MR-based partial-volume correction schemes for PET. *J. Nucl. Med.* 40 2053–2065. 10616886

[B70] Meltzer C. C. Leal J. P. Mayberg H. S. Wagner H. N. Jr. Frost J. J. (1990). Correction of PET data for partial volume effects in human cerebral cortex by MR imaging. *J. Comput. Assist. Tomogr.* 14 561–570. 10.1097/00004728-199007000-00011 2370355

[B71] Meltzer C. C. Zubieta J. K. Links J. M. Brakeman P. Stumpf M. J. Frost J. J. (1996). MR-based correction of brain PET measurements for heterogeneous gray matter radioactivity distribution. *J. Cereb. Blood Flow Metabol.* 16 650–658. 10.1097/00004647-199607000-00016 8964805

[B72] Minhas D. S. Price J. C. Laymon C. M. Becker C. R. Klunk W. E. Tudorascu D. L. (2018). Impact of partial volume correction on the regional correspondence between in vivo [C-11]PiB PET and postmortem measures of Aβ load. *Neuroimage Clin.* 19 182–189. 10.1016/j.nicl.2018.04.007 30023168 PMC6050460

[B73] Montgomery A. J. Thielemans K. Mehta M. A. Turkheimer F. Mustafovic S. Grasby P. M. (2006). Correction of head movement on PET studies: comparison of methods. *J. Nucl. Med.* 47 1936–1944. 17138736

[B74] Müller-Gärtner H. W. Links J. M. Prince J. L. Bryan R. N. McVeigh E. Leal J. P. (1992). Measurement of radiotracer concentration in brain gray matter using positron emission tomography: MRI-based correction for partial volume effects. *J. Cereb. Blood Flow Metabol.* 12 571–583. 10.1038/jcbfm.1992.81 1618936

[B75] Musafargani S. Ghosh K. K. Mishra S. Mahalakshmi P. Padmanabhan P. Gulyas B. (2018). PET/MRI: a frontier in era of complementary hybrid imaging. *Eur. J. Hybrid Imaging* 2:12. 10.1186/s41824-018-0030-6 29998214 PMC6015803

[B76] Nuyts J. (2007). “The use of mutual information and joint entropy for anatomical priors in emission tomography,” in *Proceedings of the 2007 IEEE Nuclear Science Symposium Conference Record* (Honolulu, HI: IEEE), 4149–4154. 10.1109/NSSMIC.2007.4437034

[B77] Nuyts J. Baete K. Beque D. Dupont P. (2005). Comparison between MAP and postprocessed ML for image reconstruction in emission tomography when anatomical knowledge is available. *IEEE Trans. Med. Imaging* 24 667–675. 10.1109/TMI.2005.846850 15889553

[B78] Okazawa H. Higashino Y. Tsujikawa T. Arishima H. Mori T. Kiyono Y. (2018). Noninvasive method for measurement of cerebral blood flow using O-15 water PET/MRI with ASL correlation. *Eur. J. Radiol.* 105 102–109. 10.1016/j.ejrad.2018.05.033 30017265

[B79] Olesen O. Paulsen R. Højgaard L. Roed B. Larsen R. (2012). Motion tracking for medical imaging: a nonvisible structured light tracking approach. *IEEE Trans. Med. Imaging* 31 79–87. 10.1109/tmi.2011.2165157 21859614

[B80] Ouyang J. Li Q. El Fakhri G. (2013). Magnetic resonance-based motion correction for positron emission tomography imaging. *Semin. Nucl. Med.* 43 60–67. 10.1053/j.semnuclmed.2012.08.007 23178089 PMC3508789

[B81] Ouyang X. Wong W. Johnson V. Hu X. Chen C. (1994). Incorporation of correlated structural images in PET image reconstruction. *IEEE Trans. Med. Imaging* 13 627–640. 10.1109/42.363105 18218541

[B82] Pichler B. J. Kolb A. Nägele T. Schlemmer H.-P. (2010). PET/MRI: paving the way for the next generation of clinical multimodality imaging applications. *J. Nucl. Med.* 51 333–336. 10.2967/jnumed.109.061853 20150252

[B83] Poulin E. Lebel R. Croteau E. Blanchette M. Tremblay L. Lecomte R. (2013). Conversion of arterial input functions for dual pharmacokinetic modeling using Gd-DTPA/MRI and 18F-FDG/PET. *Magn. Reson. Med.* 69 781–792. 10.1002/mrm.24318 22570280

[B84] Poulin E. Lebel R. Croteau E. Blanchette M. Tremblay L. Lecomte R. (2015). Optimization of the reference region method for dual pharmacokinetic modeling using Gd-DTPA/MRI and (18) F-FDG/PET. *Magn. Reson. Med.* 73 740–748. 10.1002/mrm.25151 24604379

[B85] Pretorius P. H. King M. A. (2009). Diminishing the impact of the partial volume effect in cardiac SPECT perfusion imaging. *Med. Phys.* 36 105–115. 10.1118/1.3031110 19235379 PMC2738604

[B86] Quarantelli M. Berkouk K. Prinster A. Landeau B. Svarer C. Balkay L. (2004). Integrated software for the analysis of brain PET/SPECT studies with partial-volume-effect correction. *J. Nucl. Med.* 45 192–201. 14960635

[B87] Queiroz M. A. Barbosa F. G. Buchpiguel C. A. Cerri G. G. (2018). Positron emission tomography/magnetic resonance imaging (PET/MRI): an update and initial experience at HC-FMUSP. *Rev. Assoc. Med. Bras.* 64 71–84. 10.1590/1806-9282.64.01.71 29561945

[B88] Rangarajan A. Hsiao I.-T. Gindi G. (2000). A bayesian joint mixture framework for the integration of anatomical information in functional image reconstruction. *J. Math. Imaging Vis.* 12 199–217. 10.1023/a:1008314015446

[B89] Reader A. J. Julyan P. J. Williams H. Hastings D. L. (2003). EM algorithm system modeling by image-space techniques for PET reconstruction. *IEEE Trans. Nucl. Sci.* 50 1392–1397. 10.1109/tns.2003.817327

[B90] Reilhac A. Merida I. Irace Z. Stephenson M. Weekes A. Chen C. (2018). Development and validation of a rebinner with rigid motion correction for the siemens PET-MR scanner: application to a large cohort of [^11^C]-PIB scans. *J. Nucl. Med*. 59 1761–1767. 10.2967/jnumed.117.206375 29653974

[B91] Rousset O. Ma Y. Evans A. (1998). Correction for partial volume effects in PET: principle and validation. *J. Nucl. Med.* 39 904–911. 9591599

[B92] Rullmann M. Dukart J. Hoffmann K. T. Luthardt J. Tiepolt S. Patt M. (2016). Partial-volume effect correction improves quantitative analysis of ^18^F-florbetaben β-amyloid PET scans. *J. Nucl. Med.* 57 198–203. 10.2967/jnumed.115.161893 26541776

[B93] Sastry S. Carson R. (1997). Multimodality bayesian algorithm for image reconstruction in positron emission tomography: a tissue composition model. *IEEE Trans. Med. Imaging* 16 750–761. 10.1109/42.650872 9533576

[B94] Sattarivand M. Kusano M. Poon I. Caldwell C. (2012). Symmetric geometric transfer matrix partial volume correction for PET imaging: principle, validation and robustness. *Phys. Med. Biol.* 57 7101–7116. 10.1088/0031-9155/57/21/7101 23051703

[B95] Scholl M. Lockhart S. N. Schonhaut D. R. O’Neil J. P. Janabi M. Ossenkoppele R. (2016). PET imaging of tau deposition in the aging human brain. *Neuron* 89 971–982. 10.1016/j.neuron.2016.01.028 26938442 PMC4779187

[B96] Schwarz C. G. Gunter J. L. Lowe V. J. Weigand S. Vemuri P. Senjem M. L. (2019). A comparison of partial volume correction techniques for measuring change in serial amyloid PET SUVR. *J. Alzheimers Dis.* 67 181–195. 10.3233/jad-180749 30475770 PMC6398556

[B97] Schwarz C. G. Senjem M. L. Gunter J. L. Tosakulwong N. Weigand S. D. Kemp B. J. (2017). Optimizing PiB-PET SUVR change-over-time measurement by a large-scale analysis of longitudinal reliability, plausibility, separability, and correlation with MMSE. *Neuroimage* 144 113–127. 10.1016/j.neuroimage.2016.08.056 27577718 PMC5183471

[B98] Shidahara M. Thomas B. A. Okamura N. Ibaraki M. Matsubara K. Oyama S. (2017). A comparison of five partial volume correction methods for tau and amyloid PET imaging with [^18^F]THK5351 and [^11^C]PIB. *Ann. Nucl. Med.* 31 563–569. 10.1007/s12149-017-1185-0 28639126

[B99] Shidahara M. Tsoumpas C. Hammers A. Boussion N. Visvikis D. Suhara T. (2009). Functional and structural synergy for resolution recovery and partial volume correction in brain PET. *Neuroimage* 44 340–348. 10.1016/j.neuroimage.2008.09.012 18852055

[B100] Smith C. T. Crawford J. L. Dang L. C. Seaman K. L. San Juan M. D. Vijay A. (2017). Partial-volume correction increases estimated dopamine D2-like receptor binding potential and reduces adult age differences. *J. Cereb. Blood Flow Metab.* 39 822–833. 10.1177/0271678x17737693 29090626 PMC6498753

[B101] Somayajula S. Panagiotou C. Rangarajan A. Li Q. Arridge S. R. Leahy R. M. (2011). PET image reconstruction using information theoretic anatomical priors. *IEEE Trans. Med. Imaging* 30 537–549. 10.1109/tmi.2010.2076827 20851790 PMC3331595

[B102] Ssali T. Anazodo U. C. Thiessen J. D. Prato F. S. St Lawrence K. (2018). A noninvasive method for quantifying cerebral blood flow by hybrid PET/MRI. *J. Nucl. Med.* 59 1329–1334. 10.2967/jnumed.117.203414 29523628

[B103] Su Y. Arbelaez A. M. Benzinger T. L. Snyder A. Z. Vlassenko A. G. Mintun M. A. (2013). Noninvasive estimation of the arterial input function in positron emission tomography imaging of cerebral blood flow. *J. Cereb. Blood Flow Metab.* 33 115–121. 10.1038/jcbfm.2012.143 23072748 PMC3597366

[B104] Su Y. Blazey T. M. Snyder A. Z. Raichle M. E. Hornbeck R. C. Aldea P. (2015a). Quantitative amyloid imaging using image-derived arterial input function. *PLoS One* 10:e0122920. 10.1371/journal.pone.0122920 25849581 PMC4388540

[B105] Su Y. Blazey T. M. Snyder A. Z. Raichle M. E. Marcus D. S. Ances B. M. (2015b). Partial volume correction in quantitative amyloid imaging. *Neuroimage* 107 55–64. 10.1016/j.neuroimage.2014.11.058 25485714 PMC4300252

[B106] Su Y. Vlassenko A. G. Couture L. E. Benzinger T. L. Snyder A. Z. Derdeyn C. P. (2017). Quantitative hemodynamic PET imaging using image-derived arterial input function and a PET/MR hybrid scanner. *J. Cereb. Blood Flow Metab.* 37 1435–1446. 10.1177/0271678x16656200 27401805 PMC5453463

[B107] Tang J. Kuwabara H. Wong D. Rahmim A. (2010). Direct 4D reconstruction of parametric images incorporating anato-functional joint entropy. *Phys. Med. Biol.* 55 4261–4272. 10.1088/0031-9155/55/15/005 20647600 PMC3104511

[B108] Tang J. Rahmim A. (2009). Bayesian pet image reconstruction incorporating anato-functional joint entropy. *Phys. Med. Biol.* 54 7063–7075. 10.1088/0031-9155/54/23/002 19904028 PMC3104509

[B109] Tang J. Rahmim A. (2015). Anatomy assisted PET image reconstruction incorporating multi-resolution joint entropy. *Phys. Med. Biol.* 60 31–48. 10.1088/0031-9155/60/1/31 25479422 PMC4489716

[B110] Thomalla G. Simonsen C. Z. Boutitie F. Andersen G. Berthezene Y. Cheng B. (2018). MRI-guided thrombolysis for stroke with unknown time of onset. *N. Engl. J. Med.* 379 611–622. 10.1056/NEJMoa1804355 29766770

[B111] Thomas B. A. Erlandsson K. Modat M. Thurfjell L. Vandenberghe R. Ourselin S. (2011). The importance of appropriate partial volume correction for PET quantification in alzheimer’s disease. *Eur. J. Nucl. Med. Mol. Imaging* 38 1104–1119. 10.1007/s00259-011-1745-9 21336694

[B112] Ullisch M. G. Scheins J. J. Weirich C. Rota Kops E. Celik A. Tellmann L. (2012). MR-based PET motion correction procedure for simultaneous MR-PET neuroimaging of human brain. *PLoS One* 7:e48149. 10.1371/journal.pone.0048149 23189127 PMC3495949

[B113] van Bergen J. M. G. Li X. Quevenco F. C. Gietl A. F. Treyer V. Meyer R. (2018). Simultaneous quantitative susceptibility mapping and flutemetamol-PET suggests local correlation of iron and beta-amyloid as an indicator of cognitive performance at high age. *Neuroimage* 174 308–316. 10.1016/j.neuroimage.2018.03.021 29548847 PMC5949258

[B114] van der Vos C. S. Koopman D. Rijnsdorp S. Arends A. J. Boellaard R. van Dalen J. A. (2017). Quantification, improvement, and harmonization of small lesion detection with state-of-the-art PET. *Eur J. Nucl. Med. Mol. Imaging* 44 4–16. (Suppl. 1), 10.1007/s00259-017-3727-z 28687866 PMC5541089

[B115] van Golen L. W. Kuijer J. P. Huisman M. C. IJzerman R. G. Barkhof F. Diamant M. (2014). Quantification of cerebral blood flow in healthy volunteers and type 1 diabetic patients: comparison of MRI arterial spin labeling and [^15^O]H_2_O positron emission tomography (PET). *J. Magn. Reson. Imaging* 40 1300–1309. 10.1002/jmri.24484 24214919

[B116] Vestergaard M. B. Lindberg U. Aachmann-Andersen N. J. Lisbjerg K. Christensen S. J. Rasmussen P. (2017). Comparison of global cerebral blood flow measured by phase-contrast mapping MRI with ^15^O-H_2_O positron emission tomography. *J. Magn. Reson. Imaging* 45 692–699. 10.1002/jmri.25442 27619317 PMC5324556

[B117] Villemagne V. L. Furumoto S. Fodero-Tavoletti M. T. Mulligan R. S. Hodges J. Harada R. (2014). In vivo evaluation of a novel tau imaging tracer for alzheimer’s disease. *Eur. J. Nucl. Med. Mol. Imaging* 41 816–826. 10.1007/s00259-013-2681-7 24514874

[B118] Vunckx K. Atre A. Baete K. Reilhac A. Deroose C. Van Laere K. (2012). Evaluation of three MRI-based anatomical priors for quantitative PET brain imaging. *IEEE Trans. Med. Imaging* 31 599–612. 10.1109/tmi.2011.2173766 22049363

[B119] Wang G. Qi J. (2015). PET image reconstruction using kernel method. *IEEE Trans. Med. Imaging* 34 61–71. 10.1109/tmi.2014.2343916 25095249 PMC4280333

[B120] Wang H. Fei B. (2012). An MR image-guided, voxel-based partial volume correction method for PET images. *Med. Phys.* 39 179–195. 10.1118/1.3665704 22225287 PMC3261055

[B121] Wehrl H. F. Sauter A. W. Divine M. R. Pichler B. J. (2015). Combined PET/MR: a technology becomes mature. *J. Nucl. Med.* 56 165–168. 10.2967/jnumed.114.150318 25593114

[B122] Yan J. Lim J. C. Townsend D. W. (2015). MRI-guided brain PET image filtering and partial volume correction. *Phys. Med. Biol.* 60 961–976. 10.1088/0031-9155/60/3/961 25575248

[B123] Yang X. Wang T. Lei Y. Higgins K. Liu T. Shim H. (2019). MRI-based attenuation correction for brain PET/MRI based on anatomic signature and machine learning. *Phys. Med. Biol.* 64:025001. 10.1088/1361-6560/aaf5e0 30524027 PMC7773209

[B124] Zahneisen B. Keating B. Ernst T. (2014). Propagation of calibration errors in prospective motion correction using external tracking. *Magn. Reson. Med.* 72 381–388. 10.1002/mrm.24943 24123287 PMC3975823

[B125] Zaidi H. Ruest T. Schoenahl F. Montandon M. (2006). Comparative assessment of statistical brain MR image segmentation algorithms and their impact on partial volume correction in PET. *Neuroimage* 32 1591–1607. 10.1016/j.neuroimage.2006.05.031 16828315

[B126] Zhu Y. Feng J. Ji J. Hou H. Chen L. Wu S. (2017a). Alteration of monoamine receptor activity and glucose metabolism in pediatric patients with anticonvulsant-induced cognitive impairment. *J. Nucl. Med.* 58 1490–1497. 10.2967/jnumed.116.189290 28302757

[B127] Zhu Y. Feng J. Wu S. Hou H. Ji J. Zhang K. (2017b). Glucose metabolic profile by visual assessment combined with statistical parametric mapping analysis in pediatric patients with epilepsy. *J. Nucl. Med.* 58 1293–1299. 10.2967/jnumed.116.187492 28104740

